# Nanoparticles in the clinic

**DOI:** 10.1002/btm2.10003

**Published:** 2016-06-03

**Authors:** Aaron C. Anselmo, Samir Mitragotri

**Affiliations:** ^1^ David H. Koch Institute for Integrative Cancer Research, Massachusetts Institute of Technology Cambridge MA 02139; ^2^ Dept. of Chemical Engineering, Center for Bioengineering University of California Santa Barbara CA 93106

**Keywords:** clinic, translational medicine, clinical translation, clinical trials, drug delivery, nanomedicine, nanoparticles

## Abstract

Nanoparticle/microparticle‐based drug delivery systems for systemic (i.e., intravenous) applications have significant advantages over their nonformulated and free drug counterparts. For example, nanoparticle systems are capable of delivering therapeutics and treating areas of the body that other delivery systems cannot reach. As such, nanoparticle drug delivery and imaging systems are one of the most investigated systems in preclinical and clinical settings. Here, we will highlight the diversity of nanoparticle types, the key advantages these systems have over their free drug counterparts, and discuss their overall potential in influencing clinical care. In particular, we will focus on current clinical trials for nanoparticle formulations that have yet to be clinically approved. Additional emphasis will be on clinically approved nanoparticle systems, both for their currently approved indications and their use in active clinical trials. Finally, we will discuss many of the often overlooked biological, technological, and study design challenges that impact the clinical success of nanoparticle delivery systems.

## 1 INTRODUCTION

Nanoparticle/microparticle delivery systems are widely investigated preclinically with many particle‐based formulations and technologies having already been introduced in the clinic.^1^, ^2^, ^3^, ^4^, ^5^ Oral, local, topical, and systemic (e.g., intravenous) administration are all proven methods that have been Food and Drug Administration (FDA)‐approved for the delivery of nanoparticles/microparticles, depending on the desired application or targeted site. For example: (a) oral delivery of particles has been approved clinically for imaging applications (e.g., Gastromark),^6^ (b) local delivery of particles has been widely used in the clinic as depot delivery systems for the extended delivery of a variety of biologics including peptides and other small molecules (e.g., DepoCyt),^4^ (c) topical application of particles has been approved clinically to increase penetration of biologics across the skin barrier (e.g., Estrasorb),^7^ and (d) systemic delivery of particles has been approved clinically for treating a variety of cancers (e.g., Doxil)^8^ and other diseases. Given the utility and success of these clinical examples, preclinical research efforts for each of these delivery methods continue to increase with particular attention placed on developing new applications and further improving their delivery and efficacy.

Of these delivery methods, intravenously administered nanoparticles receive the most attention, both preclinically and clinically. The increased interest for intravenous delivery is not surprising given that nanoparticles delivered systemically have direct access to nearly all parts of the body and thus have the most potential to influence clinical care. For this same reason, systemically delivered nanoparticles also face exceedingly difficult challenges with regards to both the delivery aspect (e.g., biological challenges)^9^, ^10^ and the regulatory aspect (e.g., study design and approval challenges).^11^, ^12^ This review focuses on the clinical translation of intravenously administered nanoparticles, with additional emphasis on the challenges faced by nanoparticles from a clinical and translational point of view. Specifically, the biological, technological, and study design challenges facing the clinical translation of nanoparticles will be discussed. Comprehensive lists of intravenous nanoparticle technologies that are either approved or currently in clinical trials will be provided to highlight the current clinical landscape.

## 2 NANOPARTICLE TYPES, APPLICATIONS, ADVANTAGES, AND POTENTIAL

**Figure 1:**
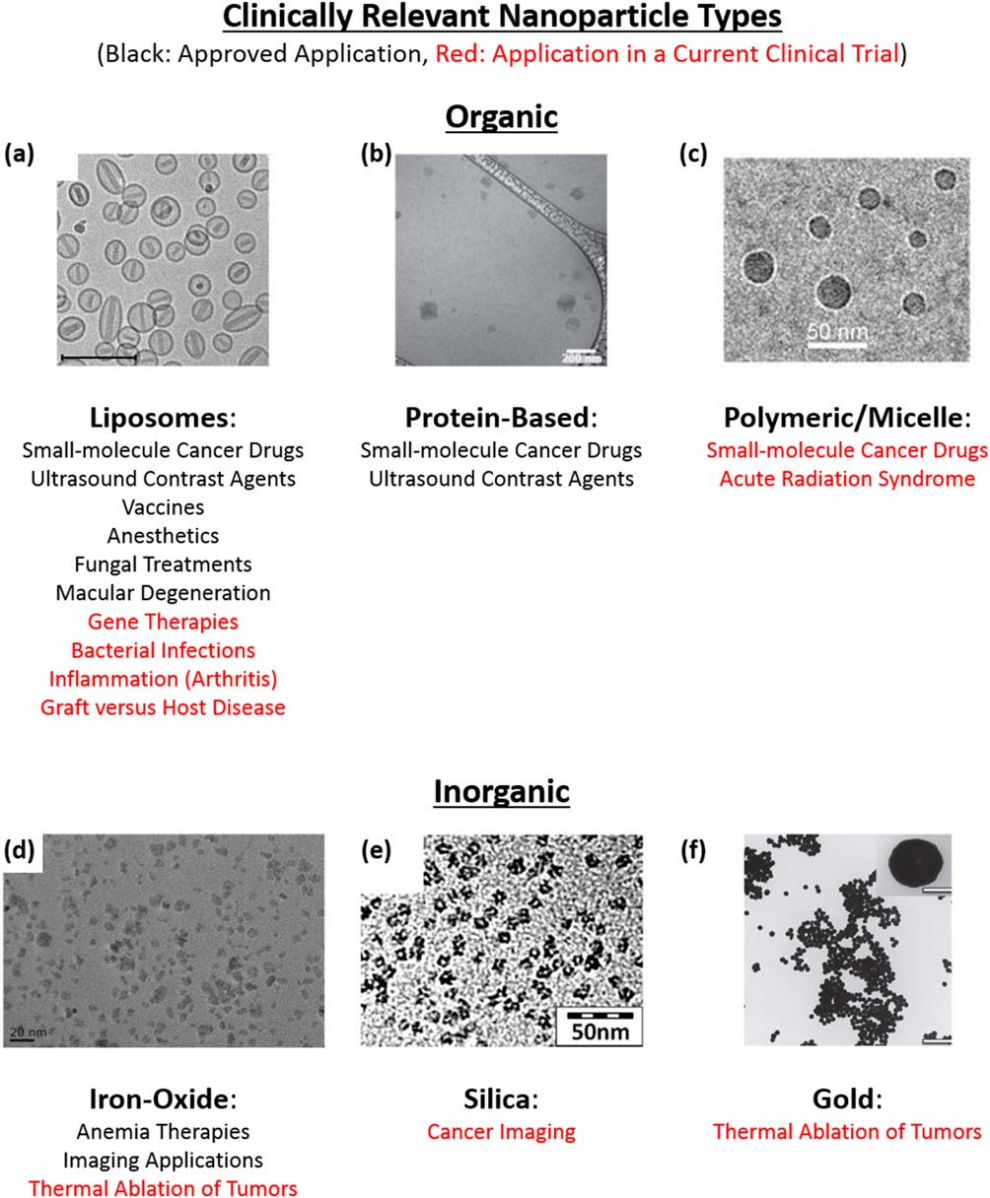
Clinically relevant nanoparticles. Organic and inorganic nanoparticles have been approved for a variety of clinical indications (black text) and are being investigated in current clinical studies for additional indications (red text). Examples included (a) Doxil (200 nm scale bar), (b) Abraxane (200 nm scale bar), (c) CRLX101 (50 nm scale bar), (d) Feraheme (20 nm scale bar), (e) early iteration of Cornell Dots (50 nm scale bar), and (f) gold nanoshells (inset: 100 nm scale bar, main figure: 1,000 nm scale bar) from Nanospectra, makers of AuroLase. (a) Reprinted from ref. 16. Copyright (2016), with permission from Elsevier. (b) Adapted by permission from Macmillan Publishers Ltd: *Nature Communications*,^17^ copyright (2015). (c) Reprinted from ref. 18 (d) Reprinted from refs. 16 and 19. Copyright (2016), with permission from Elsevier. (e) Adapted with permissions from ref. 20. Copyright (2012) American Chemical Society. (f) Reprinted from ref. 21

Therapeutic and diagnostic nanoparticles typically fall into two categories: (a) inorganic nanoparticles (e.g., gold, silica, iron oxide, etc.) and (b) organic nanoparticles (e.g., polymeric, liposomes, micelles, etc.). Inorganic nanoparticles have been successful in preclinical studies, are being developed in the clinic for a variety of applications including intraoperative sentinel lymph node imaging and thermal ablation of tumors, and have already been approved for imaging applications and anemia treatment (Figure 1).^13^, ^14^, ^15^ Alongside this, organic nanoparticles have also exhibited substantial success in the clinic where they are currently being developed for broad applications ranging from vaccination, to hemostasis, to long‐lasting depot delivery systems, to topical agents for systemic delivery through the skin.^1^, ^2^, ^3^, ^4^, ^5^ More relevant to this review are nanoparticle formulations that are delivered intravenously, and in this realm, organic nanoparticles predominantly fall into two categories: (a) nanoparticles for gene therapy applications^22^, ^23^ or (b) nanoparticles for delivery of small molecule drugs for cancer treatment (e.g., head and neck, melanoma, breast, metastatic, etc.).^24^, ^25^ Organic nanoparticle formulations for other applications (e.g., vaccines, fungal treatments, etc.) are also in development and will be highlighted here (Figure 1).

The main reasons behind the interest in nanoparticle technologies are that: (a) in the case of organic nanoparticles, they possess distinct advantages over many intravenously administered pharmaceuticals and biologics, and (b) in the case of inorganic nanoparticles, many stimuli responsive functions are possible based on specific colloidal assemblies. Organic nanoparticles can be designed and formulated to offer enhanced drug protection, controlled release, extended circulation, and improved targeting to diseased tissues as compared to their free drug counterparts.^25^, ^26^ Likewise, inorganic nanoparticles benefit from these same advantages, and additionally from stimuli‐responsive functions arising from their surface plasmon resonance (e.g., thermal heating or imaging) or magnetic responsiveness (e.g., magnetic resonance imaging [MRI] imaging or magnetic targeting) that individual drugs or other molecules (e.g., noncolloidal) do not offer.^2^, ^27^ Given these advantages, it has been a long‐held idea that nanoparticles have the potential to dramatically change clinical care by introducing new, or improving upon current, therapies. A large portion of the interest in nanoparticles stems from their potential as a platform delivery system, with the capability of exchanging specific design features (e.g., targeting antibodies, the encapsulated drug, and control over how/when the diseased site interacts with this drug) in a “plug‐and‐play” format to treat additional or other diseases.

## 3 CLINICALLY APPROVED NANOPARTICLES/MICROPARTICLES

Currently, there are a number of nanoparticle therapeutics, imaging agents, and technologies that have been approved for clinical use, either by the FDA in the United States, or the European Medicines Agency (EMA) in the European Union (Table 1). In this section, we will highlight the currently approved nanoparticles and their clinical indications.

**Table 1:** Clinically approved intravenous nanoparticle therapies and diagnostics, grouped by their broad indication

Name	Particle type/drug	Approved application/indication	Approval (year)	Investigated application/indication	ClinicalTrials.gov identifier
**Cancer Nanoparticle Medicines**					
Doxil/Caelyx (Janssen)	Liposomal doxorubicin (PEGylated)	Ovarian cancer (secondary to platinum based therapies) HIV‐associated Kaposi's sarcoma (secondary to chemotherapy) Multiple myeloma (secondary)	FDA (1995) EMA (1996)	Various cancers including: solid malignancies, ovarian, breast, leukemia, lymphomas, prostate, metastatic, or liver	166 studies mention Doxil 90 studies mention CAELYX
DaunoXome (Galen)	Liposomal daunorubicin (non‐PEGylated)	HIV‐associated Kaposi's sarcoma (primary)	FDA (1996)	Various leukemias	32 studies mention DaunoXome
Myocet (Teva UK)	Liposomal doxorubicin (non‐PEGylated)	Treatment of metastatic breast cancer (primary)	EMA (2000)	Various cancers including: breast, lymphoma, or ovarian	32 studies mention Myocet
Abraxane (Celgene)	Albumin‐particle bound paclitaxel	Advanced nonsmall cell lung cancer (surgery or radiation is not an option) Metastatic breast cancer (secondary) Metastatic pancreatic cancer (primary)	FDA (2005) EMA (2008)	Various cancers including: solid malignancies, breast, lymphomas, bladder, lung, pancreatic, head and neck, prostate, melanoma, or liver	295 studies mention Abraxane
Marqibo (Spectrum)	Liposomal vincristine (non‐PEGylated)	Philadelphia chromosome‐negative acute lymphoblastic leukemia (tertiary)	FDA (2012)	Various cancers including: lymphoma, brain, leukemia, or melanoma	23 studies mention Marqibo
MEPACT (Millennium)	Liposomal mifamurtide (non‐PEGylated)	Treatment for osteosarcoma (primary following surgery)	EMA (2009)	Osteosarcomas	4 studies mention MEPACT: 3 active/recruiting
Onivyde MM‐398 (Merrimack)	Liposomal irinotecan (PEGylated)	Metastatic pancreatic cancer (secondary)	FDA (2015)	Various cancers including: solid malignancies, breast, pancreatic, sarcomas, or brain	7 studies mention MM‐398/Onivyde: 6 active/recruiting
**Iron‐replacement nanoparticle therapies**					
CosmoFer/INFeD/Ferrisat (Pharmacosmos)	Iron dextran colloid	Iron deficient anemia	FDA (1992) Some of Europe	Iron deficient anemia	6 studies mention INFeD: 1 recruiting
DexFerrum/DexIron (American Regent)	Iron dextran colloid	Iron deficient anemia	FDA (1996)	Iron deficient anemia	6 studies mention DexFerrum
Ferrlecit (Sanofi)	Iron gluconate colloid	Iron replacement for anemia treatment in patients with chronic kidney disease	FDA (1999)	Iron deficient anemia	13 studies mention Ferrlecit: 2 recruiting
Venofer (American Regent)	Iron sucrose colloid	Iron replacement for anemia treatment in patients with chronic kidney disease	FDA (2000)	Iron deficient anemia Following autologous stem cell transplantation	44 studies mention Venofer
Feraheme (AMAG)/Rienso (Takeda)/Ferumoxytol	Iron polyglucose sorbitol carboxymethylether colloid	Iron deficiency in patients with chronic kidney disease	FDA (2009)	Iron deficient anemia Imaging: brain metastases, lymph node metastases, neuroinflammation in epilepsy, head and neck cancer, myocardial infarction, or multiple sclerosis	57 studies mention Ferumoxytol: 6 recruiting/active for anemia treatment 22 recruiting/active for imaging applications
Injectafter/Ferinject (Vifor)	Iron carboxymaltose colloid	Iron deficient anemia	FDA (2013)	Iron deficient anemia	50 studies mention Ferinject 8 studies mention Injectafer
Monofer (Pharmacosmos)	10% Iron isomaltoside 1000 colloid	Treating iron deficiency and anemia when oral methods do not work or when iron delivery is required immediately	Some of Europe	Iron deficient anemia	22 studies: 3 active/recruiting
Diafer (Pharmacosmos)	5% Iron isomaltoside 1000 colloid	Iron deficient anemia	Some of Europe	Iron deficient anemia	1 recruiting study
**Nanoparticle/microparticle imaging agents**					
Definity (Lantheus Medical Imaging)	Perflutren lipid microspheres	Ultrasound contrast agent	FDA (2001)	Ultrasound enhancement for: liver or breast or intraocular or pancreatic tumors, pulmonary diseases, heart function, transcranial injuries, strokes, or liver cirrhosis	58 studies mention Definity
Feridex I.V. (AMAG)/Endorem	Iron dextran colloid	Imaging of liver lesions	FDA (1996) Discontinued (2008)	N/A: No current studies	4 studies mention Endorem 2 studies mention Feridex No current active or recruiting studies
Optison (GE Healthcare)	Human serum albumin stabilized perflutren microspheres	Ultrasound contrast agent	FDA (1997) EMA (1998)	Ultrasound enhancement for: lymph node, renal cell carcinoma, myocardial infarction, pulmonary transit times, or heart transplant rejections	11 currently active or recruiting studies
SonoVue (Bracco Imaging)	Phospholipid stabilized microbubble	Ultrasound contrast agent	EMA (2001)	Ultrasound enhancement for: liver neoplasms, prostate or breast or pancreatic cancer, or coronary/pulmonary disease	43 studies mention SonoVue
Resovist (Bayer Schering Pharma)/Cliavist	Iron carboxydextran colloid	Imaging of liver lesions	Some of Europe Discontinued (2009)	N/A No current studies	2 studies mention Resovist: No current active or recruiting studies
Ferumoxtran‐10/Combidex/Sinerem (AMAG)	Iron dextran colloid	Imaging lymph node metastases	Only available in Holland	Imaging lymph node metastases	11 studies mention ferumoxtran‐10: 1 active
**Nanoparticle vaccines**					
Epaxal (Crucell)	Liposome with hepatitis A virus	Hepatitis A vaccine	Some of Europe (Discontinued)	Safety and immunogenicity of hepatitis A vaccine	6 studies mention Epaxal: 1 recruiting
Inflexal V (Crucell)	Liposome with trivalent‐influenza	Influenza vaccine	Some of Europe (Discontinued)	Safety and immunogenicity of influenza vaccine	14 studies mention Inflexal V: All completed
**Particle anesthetics**					
Diprivan	Liposomal propofol	Induction and maintenance of sedation or anesthesia	FDA (1989)	General anesthesia in specific situations: morbidly obese patients, open heart surgery, or spinal surgery	110 studies mention Diprivan
**Nanoparticles for fungal treatments**					
AmBisome (Gilead Sciences)	Liposomal amphotericin B	Cryptococcal Meningitis in HIV‐infected patients Aspergillus, Candida, and/or Cryptococcus species infections (secondary) Visceral leishmaniasis parasite in immunocompromised patients	FDA (1997) Most of Europe	Preventing or treating invasive fungal infections	50 studies mention AmBisome
**Nanoparticles for macular degeneration**					
Visudyne (Bausch and Lomb)	Liposomal verteporfin	Treatment of subfoveal choroidal neovascularization from age‐related macular degeneration, pathologic, or ocular histoplasmosis	FDA (2000) EMA (2000)	Macular degeneration	52 studies mention Visudyne

### 3.1 Cancer nanoparticle medicines

Many clinically approved nanoparticle formulations are used in treating various cancers at a variety of stages. Interestingly, all but one of these systems (Abraxane) is liposomal systems encapsulating an anticancer drug. Doxil, polyethylene glycol (PEG) functionalized liposomal doxorubicin, was the first approved (FDA 1995) cancer nanomedicine.^8^ Soon after, other liposomal formulations such as liposomal daunorubicin (DaunoXome),^28^ liposomal vincristine (Marqibo),^29^ and most recently liposomal irinotecan (Onivyde)^30^ were approved by the FDA, whereas non‐PEGylated liposomal doxorubicin (Myocet)^31^ and liposomal mifamurtide (MEPACT)^32^ were approved by the EMA. The lone nonliposomal nanoparticle system currently approved for cancer treatments is Abraxane, which is an albumin‐bound paclitaxel nanoparticle.^33^ The majority of these formulations are not PEGylated, with the exception of Doxil and Onivyde,^34^ which is perhaps surprising given the widely known advantages even small amounts of PEG have shown to confer to nanoparticle delivery systems.^35^, ^36^, ^37^ Additionally, all of these formulations are passively targeted, with no active or chemical‐based targeting moieties; again, this is despite the proven advantages of active‐targeting in preclinical settings.^25^, ^26^, ^38^ It is likely that the other advantages, notably their reduced toxicity stemming from their ability to preferentially accumulate at tumor sites and limit off‐target side effects via the enhanced permeation and retention (EPR) effect,^39^ are responsible for the success and increased efficacy that these approved particles have over their free drug counterparts.

### 3.2 Iron‐replacement nanoparticle therapies

Another clinical area where nanoparticles have made a significant impact is in iron‐replacement therapies for treatment of anemia (Table 1).^40^, ^41^, ^42^ In these applications, the nanoparticle (iron‐oxide colloids) is the therapeutic with the goal being to increase iron concentration in the body.^43^ These nanoparticle approaches originated from the need to address toxicity issues associated with the injection of free iron.^40^, ^42^ Using colloidal iron coated with sugars, many of these toxicity issues were resolved.^40^, ^42^ It should be noted that nanoparticles indicated for iron‐replacement undergo vastly different approval procedures, by both the FDA and EMA, as they are nonbiological complex drugs; it is a widely held belief that additional factors, stemming from their colloidal and nanoparticle nature, need to be considered during their approval (e.g., manufacturing conditions).^41^, ^44^

### 3.3 Nanoparticle/microparticle imaging agents

Alongside colloid‐based iron‐replacement therapies, similar iron‐oxide nanoparticles are clinically approved as contrast agents for MRI (Table 1).^45^, ^46^ For imaging applications, the innate magnetic responsiveness of iron‐oxide nanoparticles is used with MRI to generate contrast for imaging a variety of cancers and pathologies.^47^, ^48^ The combination of an iron‐oxide nanoparticle's MRI responsiveness and small size, which facilitates preferential uptake in tumors, provides accurate and precise imaging of cancerous tissues. Interestingly, the majority of colloidal iron‐oxide imaging agents have been discontinued in the United States and most of Europe.^13^ In addition to MRI contrast enhancers, particles can be used as intravenous ultrasound enhancing agents. In these cases, particles typically take the form of micron‐sized microbubbles.^49^, ^50^ These microbubbles provide a means to enhance contrast by stabilizing and encapsulating air bubbles, which are near‐perfect reflectors of ultrasound and would otherwise rapidly dissolve in blood if not encapsulated/formulated.^49^ Few of these products are approved and currently used in the clinic, for example, Definity (FDA approved) and SonoVue (EMA approved) are fluorocarbons or sulfur hexafluoride encased in lipid shells, respectively. Optison (FDA and EMA approved) is another ultrasound contrast agent formulated as human serum albumin encased perflutren.

### 3.4 Nanoparticles for vaccines, anesthetics, fungal treatments, and macular degeneration

Nanoparticles, or in these cases liposomes, are also used in a number of other clinical applications (Table 1). The first of these is Diprivan,^51^ which was FDA approved in 1989 as a general anesthetic.^52^ Two vaccines, Epaxal for vaccination against hepatitis A^53^ and Inflexal V for vaccination against influenza,^54^ are liposomal systems that have been approved in many European countries. Interestingly, these two vaccines use their viral glycoprotein‐liposomal template as the primary adjuvant,^55^ with Epaxal doing so in lieu of traditional adjuvants such as aluminum hydroxide.^53^ However, these vaccines have since been phased out of the clinic. In other applications, liposomes or lipid‐based nanoformulations have been clinically approved for fungal and parasitic infections. For example, the highly toxic antifungal drug amphotericin B, used for treating systemic fungal infections, has been formulated in liposomes (AmBisome).^56^ In doing so, toxicity is dramatically reduced as the pharmacokinetics and tissue distribution is improved via liposomal encapsulation. Furthermore, the liposomal formulation addresses a significant issue of the free drug form of amphotericin B, which is its insolubility in pH 7 saline. While not true liposomes, other FDA approved lipid‐complexed formulations of amphotericin B exist, such as Abelcet and Amphotec.^57^ Visudyne® is a light‐activated liposomal formulation of verteporfin. Liposomal encapsulation offers enhanced uptake in proliferating cells which particularly enhances targeting and subsequent uptake by targets neovascular areas, which, following light stimulation damages the endothelium and blocks local blood vessels to prevent and treat neovascularization.^58^

## 4 CURRENT NANOPARTICLE/MICROPARTICLE CLINICAL TRIALS

Given the successes of many of these formulations in the clinic and commercial realm, significant efforts continue to explore currently approved nanomedicines as well as developing new ones. Here, we will: (a) briefly review the current clinical trial landscape for currently approved nanoparticles (Table 1), (b) review the current clinical trial landscape regarding cutting‐edge nanoparticle formulations which are seeking approval (Table 2), and (c) highlight key technologies attempting to integrate targeting and stimuli‐responsive functions into nanoparticle delivery systems.

**Table 2:** Intravenous nanoparticle therapies and diagnostics which have not been clinically approved and are currently undergoing clinical trials (not yet recruiting, recruiting, or active), grouped by particle type as well as well as application

Name (company)	Particle type/drug	Investigated application/indication	ClinicalTrials.gov identifier (phase)
**Liposomes (cancer)**			
PROMITIL (Lipomedix Pharmaceuticals)	Pegylated liposomal mitomycin‐C	Solid tumors	NCT01705002 (Ph I)
ThermoDox® (Celsion)	Lyso‐thermosensitive liposomal doxorubicin	Temperature‐triggered doxorubicin release: Breast cancer recurrence at chest wall (microwave hypothermia) Hepatocellular carcinoma (radiofrequency ablation) Liver tumors (mild hypothermia) Refractory solid tumors (magnetic resonance high intensity focused ultrasound)	NCT00826085 (Ph I/II) NCT02112656 (Ph III) NCT02181075 (Ph I) NCT02536183 (Ph I)
VYEXOS CPX‐351 (Celator Pharmaceuticals)	Liposomal formulation of cytarabine:daunorubicin (5:1 molar ratio)	Leukemias	NCT01804101 (Not Provided) NCT02286726 (Ph II) NCT02019069 (Ph II) NCT01943682 (Ph I) NCT02269579 (Ph II) NCT02533115 (Ph IV) NCT01696084 (Ph III)
Oncoprex (Genprex)	FUS1 (TUSC2) encapsulated liposome	Lung cancer	NCT01455389 (Ph I/II)
Halaven E7389‐LF (Eisai)	Liposomal eribulin mesylate	Solid tumors	NCT01945710 (Ph I)
^188^Re‐BMEDA‐liposome	^188^Re‐N,N‐bis (2‐mercaptoethyl)‐N′,N′‐diethylethylenediamine pegylated liposome	Advanced solid tumors	NCT02271516 (Ph I)
Mitoxantrone Hydrochloride Liposome (CSPC ZhongQi Pharmaceutical Technology)	Mitoxantrone liposome	Lymphoma and breast cancer	NCT02131688 (Ph I) NCT02596373 (Ph II) NCT02595242 (Ph I) NCT02597387 (Ph II) NCT02597153 (Ph II)
JVRS‐100	Cationic liposome incorporating plasmid DNA complex for immune system stimulation	Leukemia	NCT00860522 (Ph I)
Lipocurc (SignPath Pharma)	Liposomal curcumin	Solid tumors	NCT02138955 (Ph I/II)
LiPlaCis (LiPlasome Pharma)	Liposomal formulated cisplatin with specific degradation‐controlled drug release via phospholipase A2 (PLA2)	Advanced or refractory tumors	NCT01861496 (Ph I)
MM‐302 (Merrimack Pharmaceuticals)	HER2‐targeted liposomal doxorubicin (PEGylated)	Breast cancer	NCT01304797 (Ph I) NCT02213744 (Ph II/III)
LIPUSU® (Nanjing Luye Sike Pharmaceutical Co.,Ltd.)	Paclitaxel Liposome	Advanced solid tumors, or gastric, breast cancer	NCT01994031 (Ph IV) NCT02142790 (Ph IV) NCT02163291 (Ph II) NCT02142010 (Not Provided)
**Liposomes (gene therapy: cancer)**			
TKM‐080301 (Arbutus Biopharma)	Lipid particle targeting polo‐like kinase 1 (PLK1) for delivery of siRNA	Hepatocellular carcinoma	NCT02191878 (Ph I/II)
siRNA‐EphA2‐DOPC	siRNA liposome for EphA2 knockdown	Solid tumors	NCT01591356 (Ph I)
PNT2258 (ProNAi Therapeutics)	Proprietary single‐stranded DNAi (PNT100) encapsulated in lipid nanoparticles	Lymphomas	NCT02378038 (Ph II) NCT02226965 (Ph II) NCT01733238 (Ph II)
BP1001 (Bio‐Path Holdings)	Growth factor receptor bound protein‐2 (Grb‐2) antisense oligonucleotide encapsulated in neutral liposomes	Leukemias	NCT01159028 (Ph I)
DCR‐MYC (Dicerna Pharmaceuticals)	DsiRNA lipid nanoparticle for NYC oncogene silencing	Solid tumors, multiple myeloma, lymphoma, or hepatocellular carcinoma	NCT02110563 (Ph I) NCT02314052 (Ph I/II)
Atu027 (Silence Therapeutics GmbH)	AtuRNAi liposomal formulation for PKN3 knockdown in vascular endothelium	Pancreatic cancer	NCT01808638 (Ph I/II)
SGT‐53 (SynerGene Therapeutics)	Cationic liposome with anti‐transferrin receptor antibody, encapsulating Wildtype p53 sequence	Glioblastoma, solid tumors, or pancreatic cancer	NCT02354547 (Ph I) NCT00470613 (Ph I) NCT02354547 (Ph I) NCT02340156 (Ph II)
SGT‐94 (SynerGene Therapeutics)	RB94 plasmid DNA in a liposome with anti‐transferrin receptor antibody	Solid tumors	NCT01517464 (Ph I)
MRX34 (Mirna Therapeutics)	Double‐stranded RNA mimic of miR‐34 encapsulated in liposomes	Liver cancer	NCT01829971 (Ph I)
TargomiRs (EnGeneIC)	Anti‐EGFR bispecific antibody minicells (bacteria derived nanoparticles) with a miR‐16 based microRNA payload	Mesothelioma and nonsmall cell lung cancer	NCT02369198 (Ph I)
**Liposomes (gene therapy: other)**			
ND‐L02‐s0201 (Nitto Denko)	siRNA lipid nanoparticle conjugated to Vitamin A	Hepatic fibrosis	NCT02227459 (Ph I)
ARB‐001467 TKM‐HBV (Arbutus Biopharma)	Lipid particle containing three RNAi therapeutics that target three sites on the HBV genome	Hepatitis B	NCT02631096 (Ph II)
Patisiran ALN‐TTR02 (Alnylam Pharmaceuticals)	Lipid nanoparticle RNAi for the knockdown of disease‐causing TTR protein	Transthyretin (TTR)‐mediated amyloidosis	NCT02510261 (Ph III) NCT01961921 (Ph II) NCT01960348 (Ph III)
**Liposomes (other)**			
CAL02 (Combioxin SA)	Sphingomyelin and cholesterol liposomes for toxin neutralization	Pneumonia	NCT02583373 (Ph I)
Nanocort (Enceladus in collaboration with Sun Pharma Global)	Liposomal Prednisolone (PEGylated)	Rheumatoid arthritis and hemodialysis fistula maturation	NCT02495662 (Ph II) NCT02534896 (Ph III)
RGI‐2001 (Regimmune)	Liposomal formulaton of α‐GalCer	Mitigating graft versus host disease following stem cell transplant	NCT01379209 (Ph I/II)
Sonazoid	F‐butane encapsulated in a lipid shell	Contrast enhanced ultrasound for imaging hepatocellular carcinoma, skeletal muscle perfusion, or for estimating portal hypertension	NCT00822991 (Not Provided) NCT02398266 (Ph II) NCT02188901 (Not Provided) NCT02489045 (Ph IV)
**Polymeric and micelles (cancer)**			
AZD2811 (AstraZeneca with BIND Therapeutics)	Aurora B kinase inhibitor in BIND therapeutics polymer particle accurin platform	Advanced solid tumors	NCT02579226 (Ph I)
BIND‐014 (BIND Therapeutics)	PSMA targeted (via ACUPA) docetaxel PEG‐PLGA or PLA‐PEG particle	Prostate, metastatic, nonsmall cell lung, cervical, head and neck, or KRAS positive lung cancers	NCT02479178 (Ph II) NCT02283320 (Ph II) NCT01812746 (Ph II) NCT01792479 (Ph II) NCT01300533 (Ph I)
Cynviloq IG‐001 (Sorrento)	Paclitaxel polymeric micelle nanoparticle	Breast cancer	NCT02064829 (Not Provided)
Genexol‐PM (Samyang Biopharmaceuticals)	Paclitaxel polymeric micelle nanoparticle	Head and neck or breast cancer	NCT01689194 (Ph II) NCT02263495 (Ph II) NCT00912639 (Ph IV)
NC‐6004 Nanoplatin (Nanocarrier)	Polyamino acid, PEG, and cisplatin derivative micellar nanoparticle	Advanced solid tumors, lung, biliary, bladder, or pancreatic cancers	NCT02240238 (Ph I/II) NCT02043288 (Ph III)
NC‐4016 DACH‐Platin micelle (Nanocarrier)	Polyamino acid, PEG, and oxaliplatin micellar nanoparticle	Advanced solid tumors or lymphomas	NCT01999491 (Ph I)
NK105 (Nippon Kayaku)	Paclitaxel micelle	Breast cancer	NCT01644890 (Ph III)
Docetaxel‐PM DOPNP201 (Samyang Biopharmaceuticals)	Docetaxel micelle	Head and neck cancer and advanced solid tumors	NCT02639858 (Ph II) NCT02274610 (Ph I)
CriPec (Cristal Therapeutics)	Docetaxel micelles	Solid tumors	NCT02442531 (Ph I)
CRLX101 (Cerulean)	Cyclodextrin based nanoparticle‐camptothecin conjugate	Ovarian, renal cell, small cell lung, or rectal cancers	NCT02187302 (Ph II) NCT02010567 (Ph I/II) NCT02389985 (Ph I) NCT01803269 (Ph II) NCT01652079 (Ph II)
CRLX301 (Cerulean)	Cyclodextrin based nanoparticle‐docetaxel conjugate	Dose escalation study in advanced solid tumors	NCT02380677 (Ph I/II)
**Polymeric and micelles (other)**			
RadProtect (Original BioMedicals)	PEG, iron, and amifostine micelle Transferrin‐mediated chelation for amifostine release	Dose escalation and safety for acute radiation syndrome	NCT02587442 (Ph I)
**Albumin‐bound (cancer)**			
ABI‐009 (Aadi with Celgene)	Albumin bound rapamycin	Bladder cancer, PEComa, or pulmonary arterial hypertension	NCT02009332 (Ph I/II) NCT02587325 (Ph I) NCT02494570 (Ph II)
ABI‐011 (NantBioScience)	Albumin bound thiocolchicine analog (IDN 5405)	Solid tumors or lymphomas	NCT02582827 (Ph I)
**Inorganic (Cancer)**			
AuroLase (Nanospectra Biosciences)	PEG‐coated silica‐gold nanoshells for near infrared light facilitated thermal ablation	Thermal ablation of solid primary and/or metastatic lung tumors	NCT01679470 (Not Provided)
NBTXR3 PEP503 (Nanobiotix)	Hafnium oxide nanoparticles stimulated with external radiation to enhance tumor cell death via electron production	Locally advanced squamous cell carcinoma	NCT01946867 (Ph I)
Cornell Dots	Silica nanoparticles with a NIR fluorophore, PEG coating, and a ^124^I radiolabeled cRGDY targeting peptide	Imaging of melanoma and malignant brain tumors	NCT01266096 (Not Provided)
Magnablate	Iron nanoparticles	Thermal ablation for prostate cancer	NCT02033447 (Ph 0)

### 4.1 Previously approved nanoparticles

By seeking approval for additional indications, currently approved nanoparticle systems experience a more direct path to clinical approval as compared to a newer, developing, technology. This is because already approved nanoparticles have proven their safety and efficacy in humans and, if commercialized, likely meet good manufacturing practice (GMP) standards.

#### 4.1.1 Cancer nanoparticle medicine

As cancer nanomedicines were approved by the FDA over 20 years ago, it is not surprising that these currently approved nanoparticles are investigated in the largest number of current clinical trials. For example, Doxil and Abraxane are mentioned in over 160 and 290 clinical studies, respectively. More recently approved products such as Marqibo, MEPACT, and Onivyde, also have a strong presence in clinical trials. These trials build on each individual nanoparticle's current indications by seeking approval for: (a) additional cancer types, (b) a combination therapy with other therapeutic agents, or (c) upgrading their use from a secondary therapy to a primary first‐line therapy.

#### 4.1.2 Iron‐replacement nanoparticle therapies

Of all the FDA approved iron‐replacement nanoparticle therapies, only few remain active in clinical trials. For example, CosmoFer/INFeD/Ferrisat, DexFerrum/DexIron, Ferrlecit, Monofer, and Diafer show limited activity in current clinical trials, whereas Ferinject/Injectafer, Feraheme/Rienso/Ferumoxytol, and Venofer show dramatically more activity, mostly for iron‐replacement in various clinical settings. Special attention should be placed on ferumoxytol/Feraheme/Rienso, as additional approval is being sought for a number of imaging applications which is beyond its approved indication of iron‐replacement (discussed in detail in the next section).

#### 4.1.3 Nanoparticle/microparticle imaging agents

FDA or EMA approved iron‐oxide contrast agents all show extremely low activity in current clinical trials. As stated earlier, Feridex I.V./Endorem, Resovist/Cliavist, and Combidex/Sinerem were all discontinued which is reflected by their lack of presence in current clinical trials. It is unlikely that these approved products will resurface in the clinic given that the manufacturer no longer produces them, either for clinical or research purposes. However, ferumoxytol (Feraheme or Rienso), which is approved for iron‐replacement therapies is broadly investigated for imaging applications in the clinic. Indeed, ferumoxytol is the most widely investigated iron‐oxide particle with the majority of clinical trials focused on imaging of various cancers or other pathologies (22 for imaging vs. 6 for anemia treatment). This is likely because there is a severe unmet need of iron‐oxide imaging agents in the clinical, stemming from the discontinuation of all other iron‐oxide imaging products. Approval of an iron‐oxide formulation that is already used in the clinic and also mass‐produced, is likely a more straight‐forward path to approval as opposed to a nonapproved technology. The ultrasound contrast enhancers SonoVue, Optison, and Definity are all being investigated in a number of clinical trials: 43, 11 active/recruiting, and 58, respectively. While not a currently approved indication, except for SonoVue, few of these current clinical trials are investigating microbubble use for tumor imaging applications.

#### 4.1.4 Nanoparticles for vaccines, anesthetics, fungal treatments, and macular degeneration

Epaxal and Inflexal V, approved in some European countries as liposomal‐based vaccines, are not investigated in current clinical studies, likely because they have been phased out of clinical use. In addition, the platform of intravenous virosomes developed by Crucell does not appear to be in any current clinical trials, for any vaccine. FDA‐approved Visudyne, approved for treating neovascularization is currently being investigated in clinical trials focused on combining it with other neovascularization therapies. Diprivan, FDA approved in 1989, still persists in clinical trials, mostly for approval as an anesthetic for special cases (e.g., morbidly obese patients, spinal or open‐heart surgeries). AmBisome, approved nearly two decades ago in 1997 by the FDA, is still studied in the clinic for additional bacterial/fungal infections and in tolerability and efficacy in patients with other diseases or complications.

### 4.2 Cancer nanomedicines

Cancer nanomedicines receive the most attention of all nanoparticle indications or applications in clinical trials for therapeutic purposes (e.g., nonimaging applications). This interest is built on the history and success of approved nanomedicines such as Doxil and Abraxane, which together represent the majority of all nanoparticle therapies currently in clinical trials. Here, we will discuss nanoparticle cancer therapies currently in clinical trials, with special emphasis on those that are not currently approved (Table 2).

#### 4.2.1 Gene therapy

Efforts to package and deliver siRNA or mRNA in nanoparticles for therapeutic applications, especially in cancer, are beginning to enter the clinic (Table 2).^22^, ^59^ These therapies are broadly encompassed as gene therapies, and few of these nanoparticle gene therapy trials have already been completed.^60^, ^61^ As with nanoparticles investigated for other indications and applications, the majority of these systems are liposome‐based. Few examples of these systems in current clinical trials include SGT‐53, which has shown success in restoring function of human suppressor gene p53 by delivering a plasmid containing wild‐type p53 sequence.^62^ The implications of this technology are immense, as p53 dysfunction is present in most cancers^63^ and is believed to be a requirement for tumor growth^64^; as such, technologies restoring its proper function can potentially be used to treat a number of cancers. Other current clinical studies leverage the use of proprietary engineered‐siRNAs, which show enhanced potency as compared to traditional siRNA. DCR‐MYC is a lipid nanoparticle therapy that knocks down a key oncoprotein (MYC),^65^ which is otherwise untreatable with standard therapies.^63^ As a result, DCR‐MYC may be a powerful therapy alone or in combination with more standard therapies. A liposomal siRNA formulation, Atu027, utilizes a proprietary AtuRNAi which targets and knocks down PKN3, a widely known gene causing malignant cell growth.^66^ The early clinical results are promising having shown limited cytokine activation, that it is well‐tolerated in patients, and capable of achieving disease stabilization in 41% of pancreatic cancer patients.^67^, ^68^ While many nanoparticle‐based gene therapies are in clinical trials either for gene knockdown or repair, nanoparticle‐based gene editing therapies are close behind, although at the moment, these systems are only investigated preclinically.^69^, ^70^

#### 4.2.2 Chemotherapeutics and anticancer drugs

Clinical trials focusing on encapsulating and delivering chemotherapeutics in nanoparticles are abundant (Table 2). Again, most of these systems are liposomes, with many of these liposomal systems having similar design features with already approved liposomal systems (e.g., nontargeted, PEGylated, non‐PEGylated, or encapsulating a single drug). Still, few of these clinically investigated liposomal systems are introducing novel design features in the clinic. For example, VYEXOS/CPX‐351 is a combination therapy encapsulating a synergistic ratio of two anticancer drugs (cytarabine and daunorubicin) and early clinical results have defined the recommended dose^71^ with survival advantages being shown in some patients as compared to standard chemotherapy regimens.^72^ Free drugs find combination delivery challenging, as it is straightforward to deliver exact molar ratios of two drugs to the tumor site when administered systemically, as two separate drugs will exhibit distinct pharmacokinetic and off‐site interaction profiles. However, particles ensure that the tumor receives this exact drug ratio for synergistic treatment.

Other systems investigate polymeric or micelle formulations of already established chemotherapeutics and treatments. For example, there are a number of different paclitaxel or docetaxel micelles currently in clinical trials. A broad approach, which aims to take advantage of a nanoparticle's control over circulation and biodistribution, is to target delivery of highly toxic anticancer drugs which would otherwise be too toxic in their free drug form. Delivery of the highly toxic drug camptothecin is being tested in cyclodextrin‐based nanoparticles (CRLX101), with early clinical results indicating good tolerability^73^ and tumor reduction in 74% of patients.^74^ This particular system is in five current trials and has received significant attention where numerous publications highlight its path to the clinic.^75^ In a similar approach, LiPlaCis,^76^ NC‐6004 Nanoplatin,^77^ and NC‐4016 DACH‐Platin are encapsulated forms of the wildly successful, and already approved, platinum‐based chemotherapies.^78^ The goal here is to reformulate platinum therapies as a way to avoid the serious side effects of nonencapsulated/formulating platinum drugs (e.g., kidney toxicity).^79^

A number of other nanoparticle cancer therapies are designed to treat cancer in nonstandard methods. JVRS‐100 is a cationic liposome incorporating noncoding plasmid DNA that is currently being investigated in the clinic as a means to stimulate the immune system to fight against the host's cancer. This is done via the CpG motifs contained in the DNA in combination with adjuvant effects from the liposome.^80^ JVRS‐100's efficacy has been proven in preclinical studies as a means to stimulate the immune system to fight against leukemias.^81^ Considering that nanoparticles are more often designed to avoid immune system and/or complement activation, the methodology behind delivery system is less intuitive; however, it is clear that JVRS‐100 is designed to utilize key features of nanoparticles, notably their uptake by the reticulo‐endothelial system and abilities to encapsulate and protect DNA, to act as an ideal system for immune system activation. Other systems (^188^Re‐BMEDA‐liposome) are incorporating radioactive isotopes into liposomes as a means to target the delivery of the radionuclides to tumors. A number of other novel delivery nanoparticle systems are in clinical trials for cancer therapeutics such as targeted and stimuli responsive nanoparticles systems. These targeting and stimuli‐responsive systems will be highlighted in the next sections.

### 4.3 Applications other than cancer, iron‐replacement, or imaging

Additional clinical trials are focused on testing nanoparticles for applications other than cancer, iron‐replacement, or imaging (Table 3). For example, liposomal siRNA (ARB‐001467) is being used to treat hepatitis B by knocking down three genes in the hepatitis B genome,^82^ effectively limiting antiviral resistance by facilitating knockdown of viral mRNA transcripts and antigens. In another gene therapy example, siRNA lipid nanoparticles (ND‐L02‐s0201) are being studied in clinical trials to downregulate procollagen synthesis, for the treatment of hepatic fibrosis.^83^ Alnylam is continuing their previously published work on a liposomal siRNA therapeutic for the treatment of Transthyretin (TTR)‐mediated amyloidosis by targeting knockdown of the TTR protein. Early clinical results have shown up to 94% knockdown following treatment and 77% knockdown after 28 days.^84^, ^85^ Collectively, these noncancer gene therapies are unique and potentially impactful in that they do not treat symptoms of diseases, but potentially provide a cure.

**Table 3:** Summary of current clinical trials of intravenous nanoparticles: (i) for applications other than cancer, iron‐replacement, or imaging, (ii) that are targeted to specific tissues, and (iii) that are stimuli‐responsive

Name (company)	Particle type/drug	Investigated application/indication	ClinicalTrials.gov identifier (phase)
**Applications other than cancer, iron‐replacement, or imaging**			
ND‐L02‐s0201 (Nitto Denko)	siRNA lipid nanoparticle conjugated to Vitamin A	Hepatic fibrosis	NCT02227459 (Ph I)
ARB‐001467 TKB‐HBV (Arbutus Biopharma)	Lipid particle containing three RNAi therapeutics that target three sites on the HBV genome	Hepatitis B	NCT02631096 (Ph II)
Patisiran ALN‐TTR02 (Alnylam Pharmaceuticals)	Lipid nanoparticle RNAi for the knockdown of disease‐causing TTR protein	Transthyretin (TTR)‐mediated amyloidosis	NCT02510261 (Ph III) NCT01961921 (Ph II) NCT01960348 (Ph III)
CAL02 (Combioxin SA)	Sphingomyelin and cholesterol liposomes for toxin neutralization	Pneumonia	NCT02583373 (Ph I)
Nanocort (Enceladus in collaboration with Sun Pharma Global)	Liposomal Prednisolone (PEGylated)	Rheumatoid arthritis and hemodialysis fistula maturation	NCT02495662 (Ph II) NCT02534896 (Ph III)
RGI‐2001 (Regimmune)	Liposomal formulation of α‐GalCer	Mitigating graft versus host disease following stem cell transplant	NCT01379209 (Ph I/II)
RadProtect (Original BioMedicals)	PEG, iron, and amifostine micelle Transferrin‐mediated chelation for amifostine release	Dose escalation and safety for acute radiation syndrome	NCT02587442 (Ph I)
**Targeted nanoparticles**			
ND‐L02‐s0201 (Nitto Denko)	siRNA lipid nanoparticle conjugated to Vitamin A	Hepatic fibrosis	NCT02227459 (Ph I)
BIND‐014 (BIND Therapeutics)	PSMA targeted (via ACUPA) docetaxel PEG‐PLGA or PLA‐PEG particle	Prostate, metastatic, nonsmall cell lung, cervical, head and neck, or KRAS positive lung cancers	NCT02479178 (Ph II) NCT02283320 (Ph II) NCT01812746 (Ph II) NCT01792479 (Ph II) NCT01300533 (Ph I)
MM‐302 (Merrimack Pharmaceuticals)	HER2‐targeted liposomal doxorubicin (PEGylated)	Breast cancer	NCT01304797 (Ph I) NCT02213744 (Ph II/III)
TargomiRs (EnGeneIC)	Anti‐EGFR bispecific antibody minicells (bacteria derived nanoparticles) with a miR‐16 based microRNA payload	Mesothelioma and nonsmall cell lung cancer	NCT02369198 (Ph I)
SGT‐53 (SynerGene Therapeutics)	Cationic liposome with anti‐transferrin receptor antibody, encapsulating Wildtype p53 sequence	Glioblastoma, solid tumors, or pancreatic cancer	NCT02354547 (Ph I) NCT00470613 (Ph I) NCT02354547 (Ph I) NCT02340156 (Ph II)
SGT‐94 (SynerGene Therapeutics)	RB94 plasmid DNA in a liposome with anti‐transferrin receptor antibody	Solid tumors	NCT01517464 (Ph I)
Cornell Dots	Silica nanoparticles with a NIR fluorophore, PEG coating, and a ^124^I radiolabeled cRGDY targeting peptide	Imaging of melanoma and malignant brain tumors	NCT01266096 (Not Provided)
**Stimuli responsive (nonimaging applications)**			
ThermoDox® (Celsion)	Lyso‐thermosensitive liposomal doxorubicin	Temperature‐triggered doxorubicin release: Breast cancer recurrence at chest wall (microwave hypothermia) Hepatocellular carcinoma (radiofrequency ablation) Liver tumors (mild hypothermia) Refractory solid tumors (magnetic resonance high intensity focused ultrasound)	NCT00826085 (Ph I/II) NCT02112656 (Ph III) NCT02181075 (Ph I) NCT02536183 (Ph I)
RadProtect (Original BioMedicals)	PEG, iron, and amifostine micelle Transferrin‐mediated chelation for amifostine release	Dose escalation and safety for acute radiation syndrome	NCT02587442 (Ph I)
LiPlaCis (LiPlasome Pharma)	Liposomal formulated cisplatin with specific degradation‐controlled drug release via phospholipase A2 (PLA2)	Advanced or refractory tumors	NCT01861496 (Ph I)
AuroLase (Nanospectra Biosciences)	PEG‐coated silica‐gold nanoshells for near infrared light facilitated thermal ablation	Thermal ablation of solid primary and/or metastatic lung tumors	NCT01679470 (Not Provided)
NBTXR3 PEP503 (Nanobiotix)	Hafnium oxide nanoparticles stimulated with external radiation to enhance tumor cell death via electron production	Locally advanced squamous cell carcinoma	NCT01946867 (Ph I)
Magnablate	Iron nanoparticles	Thermal ablation for prostate cancer	NCT02033447 (Ph 0)

Other liposomal nanoparticle formulations (CAL02) are designed to be a broad antitoxin therapy, currently in a clinical trial for bacterial pneumonia, by competing with host cells for toxin binding.^86^ Regimmune is developing a liposomal formulation of α‐GalCer (RGI‐2001) to induce T‐cells while maintaining other normal immune cell functions, thereby limiting graft‐versus‐host disease complications^87^ following stem cell transplantation. Enceladus is developing liposomal formulated prednisolone (Nanocort) for broad treatment of acute inflammation. RadProtect utilizes micelle nanoparticles to confer radiation protection via release of the cytoprotective drug amifostine, and is being developed by Original BioMedicals.^88^ Overall, these examples highlight the potential that nanoparticles have not only in cancer and imaging applications, but as a delivery system for a variety of clinical applications.

### 4.4 Advanced nanoparticle systems

While the majority of nanoparticle delivery systems in clinical trials build on technologies that are long‐established in their clinical utility (e.g., liposomes) or are already approved (e.g., Abraxane), some introduce aspects which are standard in academic and preclinical settings. Here, we will highlight systems that are moving nontraditional clinical nanoparticle formulations forward; specifically, nanoparticle systems that leverage chemical‐targeting or stimuli‐responsive therapeutic release (Table 3).

#### 4.4.1 Targeted delivery systems

**Figure 2:**
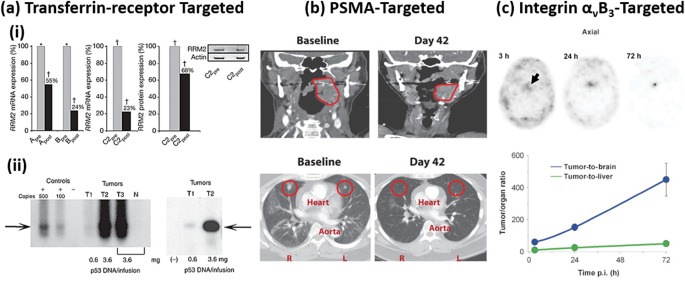
Examples of successful nanoparticle targeting in humans. (a) (i) Transferrin‐receptor targeting of a cyclodextrin‐based nanoparticle for the successful delivery of siRNA and subsequent knockdown of the anticancer target RRM2. Data show knockdown percentages of RRM2 in three patients (before: grey bars, after: black bars) as analyzed by quantitative reverse‐transcriptase polymerase chain reaction (qRT‐PCR) and western blot analysis. (ii) Transferrin‐receptor targeting of a liposomal nanoparticle for delivery of p53 for restoring p53 function. Data show increased presence of p53 in patient's tumors (as compared to negative control skin biopsy in same patients) following the targeted therapy. (b) PSMA‐targeted polymeric particles show shrinkage of tumors after two treatment cycles at 42 days for patients with tonsillar cancer (top) and lung metastases (bottom). (c) cRGDY‐peptide functionalized silica particles with radioactive iodine and a fluorescent dye (Cornell Dots) increased contrast in a pituitary lesion. Interestingly, contrast increased over time where tumor‐to‐background (both tumor‐to‐brain and tumor‐to‐liver) ratios highlight the efficiency and success of cRGDY targeting. (ai) Adapted by permission from Macmillan Publishers Ltd: *Nature*,^61^ Copyright (2010). (aii) Adapted by permission from Macmillan Publishers Ltd: *Molecular Therapy*,^62^ Copyright (2013). (b) From ref. 91. Reprinted with permission from AAAS. (c) From ref. 92. Reprinted with permission from AAAS

Interestingly, while nanoparticles and targeting antibodies are both approved for clinical use, systems combining these two technologies are lacking in both approved products and in clinical trials (Table 3).^89^ Considering the substantial preclinical research efforts into, and the proven benefits of, actively targeted nanoparticle drug delivery systems,^24^, ^25^, ^38^, ^90^ this is somewhat surprising. Still, few technologies are being investigated in the clinic using chemically targeted means to enhance delivery of a number of therapeutics. Particular attention should be placed on Calando Pharmaceutical's CALAA01 siRNA formulation, which was the first example of a targeted nanoparticle formulation for siRNA delivery.^60^, ^61^ This system used a cyclodextrin‐based particle which contained PEG, an siRNA designed to knockdown RRM2, and a transferrin receptor targeting protein, which when formulated together in a single nanoparticle was successful in achieving knockdown of the protein RRM2 (Figure 2ai).^60^, ^61^ A similar strategy utilizing transferrin targeting is being investigated by SynerGene Therapeutics (SGT‐53) to deliver plasmid containing wild‐type p53 sequence. Early clinical data show success of this targeted system in restoring the function and presence of human suppressor gene p53 in tumors (Figure 2aii).^62^ This same targeting method is used in another SynerGene Therapeutics' formulation (SGT‐94), which is an RB94‐loaded liposome. Other examples include Vitamin A conjugation to liposomes (ND‐L02‐s0201) to target delivery of siRNA to stellate cells in the liver to treat hepatic fibrosis.^83^ A HER‐2 targeted PEGylated liposomal doxorubicin (MM‐302) for treatment of breast cancer is also being developed.^93^ This builds on the success of FDA‐approved and HER‐2 targeted monoclonal antibody Herceptin (trastuzumab)^94^, ^95^ and the more recently approved antibody drug conjugate version (Kadcyla).^96^, ^97^ Other systems use bi‐specific antibodies to target delivery of mRNA therapeutics (TargomiRs).

BIND Therapeutics and the Accurin platform targets the established tumor antigen prostate‐specific membrane antigen (PSMA) for delivery of docetaxel in a PEG‐PLGA/PLA‐PEG nanoparticle (BIND‐014).^91^ These particles were screened and selected from a library of over 100 formulations to optimize their physicochemical properties. The early clinical results highlight the efficacy of BIND‐014 in shrinking tumors, even in tumors that typically show minimal response to docetaxel (Figure 2b).^91^ In another recent example, a small integrin‐targeting cRGDY peptide was used (Cornell Dots) to increase overall targeting and binding to vascular tumor markers to enhance imaging of pathological tissues including liver tumors, pituitary lesions (Figure 2c), and drug‐induced nephrotoxicity.^92^

#### 4.4.2 Stimuli‐responsive nanoparticles (nonimaging applications)

The majority of stimuli‐responsive nanoparticles are inorganic systems used for imaging applications (Table 3). However, inorganic nanoparticles made from materials such as gold or iron oxide can be used for other stimuli responsive functions, such as thermal heating or magnetic control. AuroLase, a gold nanoparticle designed to absorb light to thermally ablate solid tumors, is in a current trial as a site‐selective therapy for treatment of primary or metastatic lung tumors. Given that no active drug is used, and AuroLase is externally activated at the target site, this specific therapy has the potential for significantly reduced side effects. These efforts build on extensive preclinical testing,^98^, ^99^, ^100^ with early clinical results pointing to excellent tolerability in humans.^101^ Magnablate, an iron‐oxide nanoparticle, is being developed for similar application, except that magnetic fields are used to stimulate the nanoparticles for thermal ablation. Hafnium oxide nanoparticles (NBTXR3), developed by Nanobiotix, utilize an external radiation source to enhance cell death at the radiation site via release of electrons. In preclinical animal models, NBTXR3 showed antitumor effects with similar to standard radiation therapies^102^ and early clinical results suggest a good safety profile in humans as well as encouraging antitumor results.^103^ As these therapies attack cancer cells via a physical mechanism, it is likely that they will benefit from synergistic pairings with other, more chemical‐based, treatments.

Nanoparticle systems based on organic component can also be designed to be stimuli‐responsive. Building on the success of Doxil, Celsion Corporation is currently developing ThermoDox® as a temperature‐sensitive version of liposomal doxorubicin. ThermoDox is a heat‐sensitive liposome that release doxorubicin upon exposure to high temperatures (∼42°C). Clinical trials for ThermoDox® highlight tolerability in breast cancer patients.^104^ Additional clinical studies are underway for the treatment of hepatocellular carcinoma, breast cancer at the chest wall, and liver metastases (Table 3). In these studies, increased local temperature is achieved via microwave hypothermia, ultrasound, or radiofrequency thermal ablation. While these highlighted inorganic systems respond to external stimuli such as near infrared (NIR) lasers or magnets or radiation sources, other stimuli‐responsive systems take advantage of unique or key biological conditions or situations to control drug release. Two other clinically investigated particles, LiPlaCis and RadProtect, respond to local biological cues to release their therapeutic. In the case of LiPlaCis, the liposomes degrade more rapidly in presence of phospholipase A2,^105^ which is more abundant in tumor tissues^106^; thus, the cisplatin payload is released only in the tumor and only in the target cells. RadProtect is a micelle linked by ferrous iron which chelates with transferrin for release of amifostine in the bloodstream. While amifostine release is not linked to uptake in a target cell, this method can be used to control the rate of amifostine release into the blood.

## 5 THE MAIN CHALLENGES

Each individual nanoparticle formulation will face unique challenges in their clinical translation yet the majority of nanomedicines will encounter many of the same challenges. These challenges are biological, technological, and study design related. Here, we will focus on key challenges the majority of intravenous nanoparticle formulations face and how these challenges present unique issues from a clinical and translational point of view.

### 5.1 Biological challenges

Biological challenges including modulating biodistribution or controlling passage of nanoparticles across biological barriers and into target cells limit the effectiveness of all nanoparticle formulations. As many preclinical studies and academic groups predominately focus on these challenges, we will not review them in detail, as it has been done previously.^11^, ^107^, ^108^, ^109^ Here, we will focus on how clinically approved and clinically investigated nanomedicines are addressing these challenges and additionally highlight alternative clinical technologies (e.g., FDA‐approved targeting antibodies) that complement nanoparticle delivery systems. Additional focus will be placed on how both differences in animal and human diseases, and human disease heterogeneity, influence preclinical and clinical nanomedicine efforts.

#### 5.1.1 Biodistribution modulation

One of the main challenges facing the clinical translation of nanomedicines is controlling their biological fate (e.g., increasing target site accumulation and decreasing off‐target site accumulation). Many of the current approved and clinically investigated nanoparticles are PEGylated or PEG‐terminated which limits interactions with, and rapid clearance by, immune cells.^10^, ^37^ In doing so, nanoparticles can remain in circulation for longer periods of time and increase their chances of reaching and entering target sites (e.g., tumors via EPR effect).^39^, ^110^, ^111^ Similar effects can be achieved using hydrophilic sugar (e.g., dextran^10^) coatings as in the case of many clinically approved iron‐oxide nanoparticles. Another strategy used in the clinical (e.g., Abraxane) leverages biological proteins (e.g., human serum albumin), which likely extends the time before immune recognition as they naturally have a long circulation time.^112^ Other than PEG, sugars, or serum proteins, very few other circulation‐extending strategies have been able to successfully enter the clinic as an approved product, despite breakthroughs in preclinical academic studies.^113^

Regarding nonapproved nanoparticles in clinical studies, one particular formulation (Cornell Dots) using integrin‐targeting RGD‐based peptides for increasing tumor accumulation for imaging applications, may point toward a nanoparticle future based on personalized medicine. Building on this, preclinical peptides are routinely discovered via phage displayed, especially other similar RGD‐based peptides,^114^, ^115^ which may be a highly relevant strategy to discover either individually unique or disease‐specific peptides in humans.^116^ Currently, the most investigated attempts to modulate biodistribution in nonapproved particles introduce antibody targeting (e.g., HER2, EGFR, or transferrin receptor targeting). If successful, these systems will represent the first clinically approved examples of antibody targeted nanoparticles, which will likely facilitate the clinical investigation of additional targeted systems. A significant number of antibody therapeutics, including antibody drug‐conjugates,^117^ are approved and these specific examples may be a relevant starting point for the development of additional antibody‐targeted nanoparticle systems.

#### 5.1.2 Biological barrier breaching

Hand‐in‐hand with biodistribution and controlling nanoparticle fate is mediating their interactions with biological barriers at these target sites. The main two strategies for approved nanomedicines in the clinic include using particles of specific sizes to increase accumulation of particles in tumor sites via the EPR effect or using liposomal systems that exhibit significantly enhanced binding and uptake into target cells. Antibody targeting tested in nonapproved particles, and as highlighted above, additionally confers advantages to internalization and crossing of biological barriers, especially in the case of transferrin targeted nanoparticles. Interestingly, recent preclinical work has highlighted advantages of PEGylated particles beyond extended circulation, notably their diffusion in tissues^118^ and their abilities to pass through tight biological barriers.^36^, ^119^ It is possible that clinically used PEGylated particles have benefitted from these effects. Still, efforts to further enhance barrier breaching and tissue penetration of nanoparticles should be considered,^120^ as it is widely accepted that nanoparticle payloads are more efficacious when distributed through pathological tissues, as opposed to just the periphery.

#### 5.1.3 Heterogeneity of human disease and relevant animal models

A major issue with the clinical translation of nanoparticles is the division between preclinical studies in animals and clinical studies in humans. An example of this is the EPR effect, which has certainly been established in small animal models, however, similar evidence is lacking for humans.^121^ Other issues arising from issues relating human and animal experiments include optimization of targeting ligands, where optimization of the number of targeting ligands on a nanoparticle may not correlate between small animals and humans, or even between two humans. Currently, approved particles do not directly address these issues; however, taking a close look at similarities between approved, and nonapproved clinically investigated, formulations it is clear that methods that have been shown to work broadly (e.g., hydrophilic coatings such as PEG or sugars) are the most used. This is likely because the mechanism that PEG uses to enhance circulation is dependent on physical interactions that are common across all species/individuals (e.g., limiting opsonization), and less on biological interactions (e.g., receptor‐ligand binding), which will be impacted by disease heterogeneity. The complexities associated with species‐barrier issues are further complicated when considering the heterogeneity of human disease.^122^, ^123^ Fortunately, efforts to correlate human and small animal data are increasing given the increasing number of clinical trials.^18^, ^91^, ^124^

#### 5.1.4 The interplay between biological challenges

**Figure 3:**
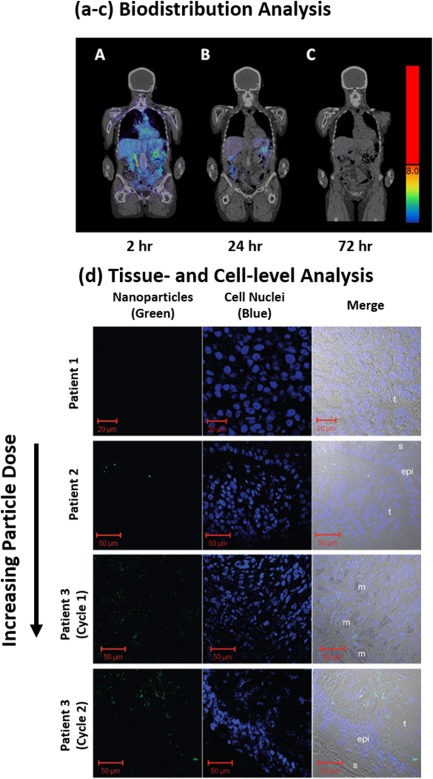
Biological challenges that intravenous nanoparticle formulations face. (a–c) Time‐dependent biodistribution [(a) 2, (b) 24, and (c) 72 hr] of Cornell Dots in a human. (d) Tissue‐ and cell‐level nanoparticle (green) confocal images of CALAA‐01 in three human patients with melanoma (epi, epidermis; m, melanophage; s, skin side; t, tumor side). (a–c) From ref. 92. Reprinted with permission from AAAS. (d) Adapted by permission from Macmillan Publishers Ltd: *Nature*,^61^ Copyright (2010)

The issues of human disease heterogeneity and the general discrepancies between animals and humans become connected and further amplified by the limitations in analyzing their biological performance (e.g., biodistribution and organ‐ and tissue‐level distribution). For example, methods to analyze, and thereby quantitatively determine, the biological fate of nanoparticles in humans is exceedingly difficult given that the most quantitative techniques require organ isolation or tissue harvesting. However, certain technologies are better designed to handle these issues (e.g., Cornell Dots), where time‐dependent biodistribution and subsequently organ‐specific accumulations in specific patients can be determined and analyzed (Figures 3a–3c).^92^ Unfortunately, nanoparticles not designed for imaging will find it difficult to achieve understanding of their biological fate. In these cases, other methods such as tissue biopsies (Figure 3d)^61^ can be used to determine the extent of penetration across and into tumor or other pathological barriers. Overall, the majority of nanoparticles cannot be detected or analyzed in such a way; unfortunately, this will not only limit their analysis and interpretation of results, but it can also limit the translation of many would be successful therapies as it may not be possible to describe, and subsequently improve, clinical failures.

### 5.2 Technological challenges

Methods to address key technological challenges of nanoparticles such as scaled‐up synthesis, performance optimization, and performance predictions will be essential in ensuring the clinical success of future nanoparticle formulations. Here, we will focus on how the limits of current nanoparticle synthesis, experimental, and prediction technologies impact and influence their clinical success and integration.

#### 5.2.1 Scale‐up synthesis

Clinical translation and integration relies on a consistent and reproducible product. With few exceptions, nanoparticles used in preclinical studies are almost exclusively synthesized in small batches and their scale‐up for large quantity synthesis is not always possible, even for clinical studies. Given the complexities in human disease, it is essential to have a consistent and highly reproducible formulation prior to the clinical trial stage. Synthesis issues have recently been encountered in the clinic, as a shortage of Doxil led to expedited approval and use of what was reported to be a generic but equivalent Doxil formulation, LipoDox.^125^ However, efficacy differences in these two formulations were reported,^126^ despite assurance that the products were identical. While it is unclear what exactly led to these differences, it is likely that reliable synthesis and scale‐up methods played a part.

#### 5.2.2 High‐throughput nanoparticle optimization

**Figure 4:**
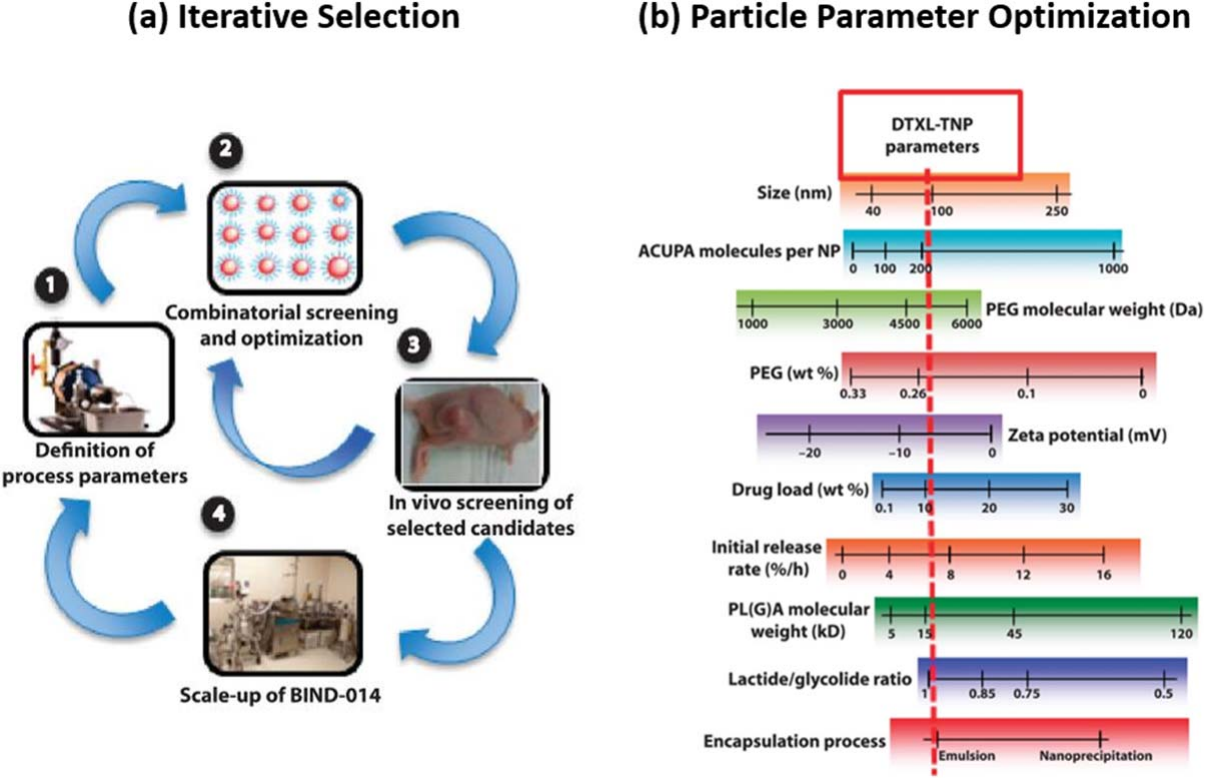
Technological challenges that intravenous nanoparticle formulations face. (a) Overview of a nanoparticle selection process based on (1) a given synthesis approach for (2) high‐throughput iterative in vitro and (3) in vivo selection of nanoparticles with favorable performance, and (4) the scale‐up of a final nanoparticle formulation. (b) A close‐up snapshot of the various particle parameters that can be iteratively optimized for a desired performance standard. (a, b) From ref. 91. Reprinted with permission from AAAS

As expected, the nanoparticle formulations offering the best performance in animal models, and thus the most potential, are the likely candidates for human translation and clinical trials. Unfortunately, the preclinical studies to determine the lead clinical candidates are for the most part not systematically designed optimized; often, formulations that are used in clinical trials are one of a handful of those investigated. Recent research efforts have described methods to test numerous nanoparticle formulations for specific biological functions or in vitro release profiles, and through selective iterations arriving at a single optimized formulation for a single specific function (Figure 4a).^91^, ^127^, ^128^ The most advanced of these strategies have proven to yield “hits” that have exhibited success in humans by optimizing specific nanoparticle parameters (Figure 4b).^91^ While this should not be introduced in the clinic or for direct human testing, efforts to systematically optimize nanoparticles preclinically will likely yield better performance clinical trials.^91^

#### 5.2.3 Predicting nanoparticle efficacy and performance

Methods to predict nanoparticle performance (e.g., computational or theoretical modeling) can be leveraged to better predict clinical trial results. Combining these techniques with experimental results and devices designed to mimic physiological tissues and conditions (e.g., organs‐on‐chips) may one day improve nanoparticle predictions of efficacy and performance.^129^ Unfortunately, this remains a challenge even at the preclinical level where relevant estimates are typically generated from compartmental analyses or pharmacokinetic models.^130^ As discussed above, correlation between human and animal data is essential. Early efforts to correlate animal data to human clinical data have shown agreement in some aspects (e.g., pharmacokinetics) but disagreement in other aspects (e.g., kidney toxicity).^124^ As such, these differences and similarities must be considered on a case‐by‐case basis, however, efforts to correlate results should be implemented as soon as possible so general trends, if any, can be established.

### 5.3 Study‐design challenges

In contrast to the previously discussed issues are challenges relating to approval and study design in humans. Specifically, study size and the timing of nanoparticle therapies in a treatment regimen impact how results from clinical studies are perceived. As such, clinical results greatly influence future nanoparticle clinical studies; special attention must be given to ensure that clinical trials are designed to extract the most information regarding nanoparticle interactions, fate, and function while still testing key hypotheses.

#### 5.3.1 *N* = 1 Clinical studies: Personalized medicine

As nanomedicine and personalized medicine efforts move forward, *N* = 1 clinical studies will be required to move toward a system capable of considering individual variability.^131^ Many factors including genetic, environmental, and past and current treatments influence medicine efficacy. Perhaps surprisingly, many approved medications do not provide benefits to all who take them and this issue stems from original clinical study design that often overlooks trends in outliers which can affect a specific subset of patients.^131^ Importantly, data regarding whether a patient either responds, or does not respond, to a given a treatment need to be collected, analyzed, and made available. Nanomedicines provide a direct method to fine tune physicochemical properties on an individual basis that can tip the balance regarding a patient responding or not responding; however, to leverage these inherent advantages of nanoparticles, correlations between patients who either respond well or poorly and their previous medical history must be made. For example, prior medical history of all patients should be well‐documented so as to determine groups which respond better to treatments in trials.

#### 5.3.2 Clinical trials as first‐line therapies

The introduction of therapeutic nanoparticles in a treatment regimen during clinical trials is rarely, if ever, a first‐line therapy. Typically, the only instances where this is the case is for established and approved nanoparticle systems (e.g., Doxil or Abraxane). Given that most of these therapies are not established, and in many cases their tolerability is not known, it is safest and most appropriate to investigate their efficacy as a last resort. As such, it is often the case that clinical trials are only available to patients who have stopped responding to treatments, as is the case with many cancer patients. While this represents a grand clinical challenge (i.e., treating or curing what is labeled a terminal disease), it may skew the potential of a nanoparticle therapy to benefit those who are likely still treatable.

#### 5.3.3 Studies extracting fundamental information

Empirical results are often the standard for preclinical studies. Indeed, it is similarly often the case that empirical results determine the commercial and clinical fate of nanoparticles in clinical trials; meaning, a binary result of improved survival or efficacy is enough to halt further investigations for a particular formulation. Indeed, lack of increased efficacy is unacceptable and in most of these cases this direction should be taken, however, these studies should be designed such that they generate of knowledge in regards to fundamental nanoparticle interactions. For example, as highlighted earlier, nanoparticles capable of facilitating data collection for time‐dependent biodistribution (Figures 3a–3c) and tissue level accumulation and distribution (Figure 3d) are better positioned to describe the empirical efficacy results, and thus, can point towards technological areas of improvement.

## 6 CONCLUSION

Many nanoparticle systems have been approved by either the FDA or EMA and are used in the clinic to either treat or diagnose disease. Significant efforts are pushing these same technologies further, by seeking approval for additional indications to impact clinical care even more. Beyond these efforts, there are a large number of clinical trials investigating novel nanoparticle systems that are, in some ways, more advanced that what has already been approved. For example, many of the nanoparticle systems in clinical trials use active targeting mechanisms to improve biodistribution or use stimuli‐responsive mechanisms to control drug release in specific areas of the body. Both of these functions are not available from any of the currently approved nanoparticle systems. This progress in the clinic over the past 20 years (since Doxil's approval) has been made possible by the extensive efforts in preclinical, commercial, and clinical studies. Furthermore, the overall outlook of nanoparticle drug delivery systems is promising, as they are also being developed for treating and curing not only cancer, but a large number of other diseases.
